# Bifurcated hepatocaval stent reconstruction for treatment of hepatic venous outflow obstruction in orthotopic liver transplantation

**DOI:** 10.1186/s42155-025-00590-7

**Published:** 2025-08-29

**Authors:** Jonathan C. Giang, Dillon M. Brown, Jeffrey Forris Beecham Chick, Eric J. Monroe, David S. Shin, Mina S. Makary

**Affiliations:** 1 Northeast Ohio Medical University, Rootstown, OH 44272 USA; 2 Section of Vascular and Interventional Radiology, Department of Radiology, University of Washington, Seattle, WA 98195 USA; 3 Section of Interventional Radiology, Department of Radiology, Seattle Children’s Hospital, University of Washington, 4800 Sand Point Way Northeast, Seattle, WA 98105 USA; 4 Division of Vascular and Interventional Radiology, Department of Radiology, The Ohio State University Wexner Medical Center, 395 W 12th Ave, 4th Floor Faculty Office Tower, Columbus, OH 43210 USA

To the Editor:

Hepatic venous outflow obstruction (HVOO) complicates 1–6% of orthotopic liver transplants (OLT), with incidence depending on the type of surgical anastomosis [1]. This condition manifests as congestive hepatopathy and post-hepatic portal hypertension, potentially leading to graft failure and death if untreated. Current management is balloon angioplasty with optional stent placement, yet conventional inferior vena cava (IVC) and hepatic vein (HV) stenting carries risks of adverse events. In particular, stent migration and stent fracture may cause life-threatening venous or cardiopulmonary injury [1]. Moreover, solitary stents at the IVC and HV confluence may lead to recurrent HVOO through mechanical obstruction and endothelial hyperplasia [1]. Bifurcated stent reconstruction techniques have been previously described in the literature by Aaberg et al. [2]. The purpose of this study was to report on the efficacy and safety of bifurcated hepatocaval stent reconstruction for treatment of anastomotic stenoses in OLT. This stenting technique entailed placing interlocking stents in a “T-shaped” configuration at the IVC and HV confluence (Figs. 1 and 2).

**Fig. 1.**
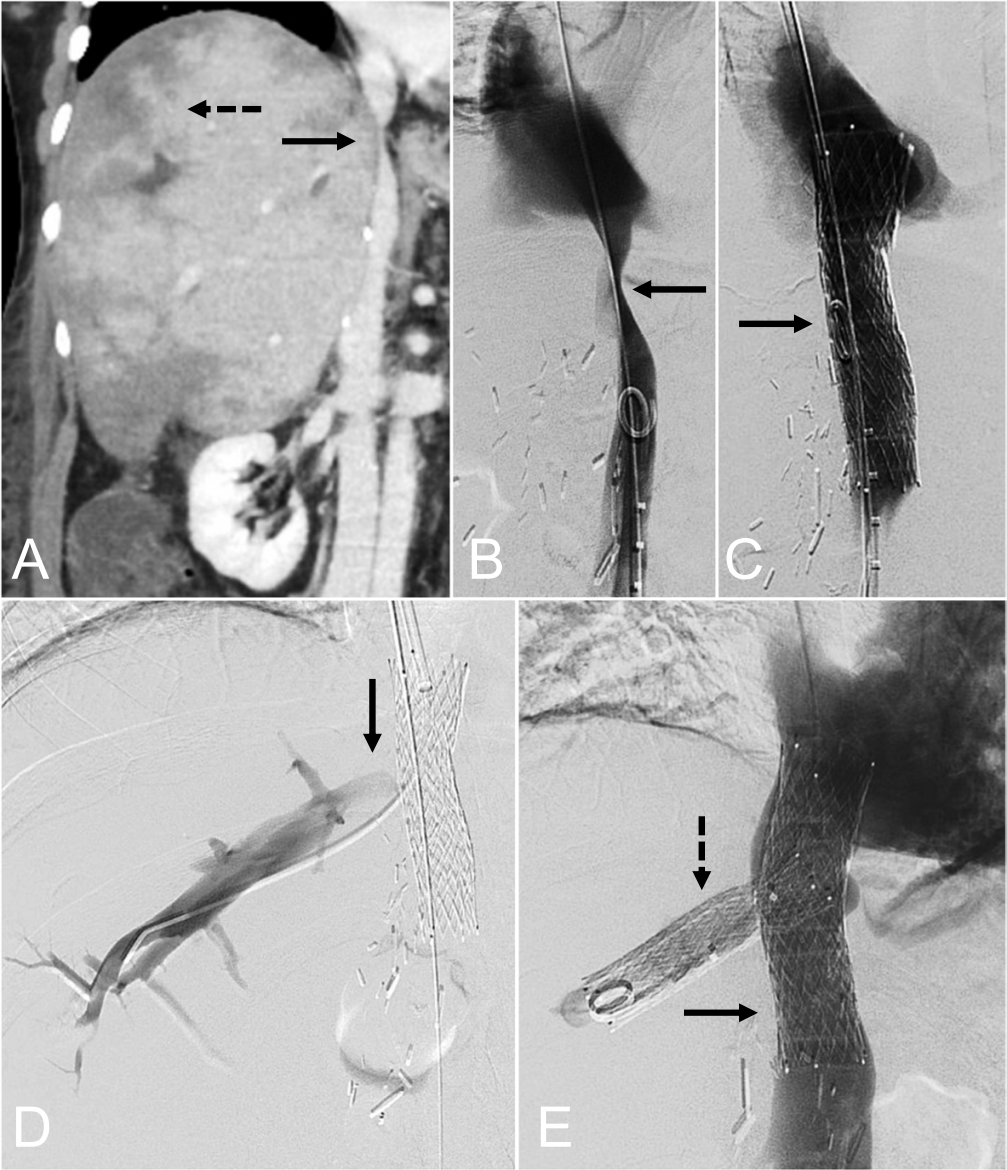
69-year-old male with alcoholic cirrhosis and HCC requiring orthotopic liver transplantation with piggyback anastomosis presented with ascites and transaminitis, and found to have IVC stenosis [solid arrow] resulting in outflow obstruction and hepatic congestion [dotted arrow] on contrast-enhanced CT (**A**). Diagnostic venography further confirmed IVC stenosis (**B**), which resolved with stenting utilizing a Wallstent (**C**). Further selection of the right hepatic vein through the IVC stent was achieved (**D**), and a right hepatic vein Wallstent was placed through the primary stent (**E**)

**Fig. 2.**
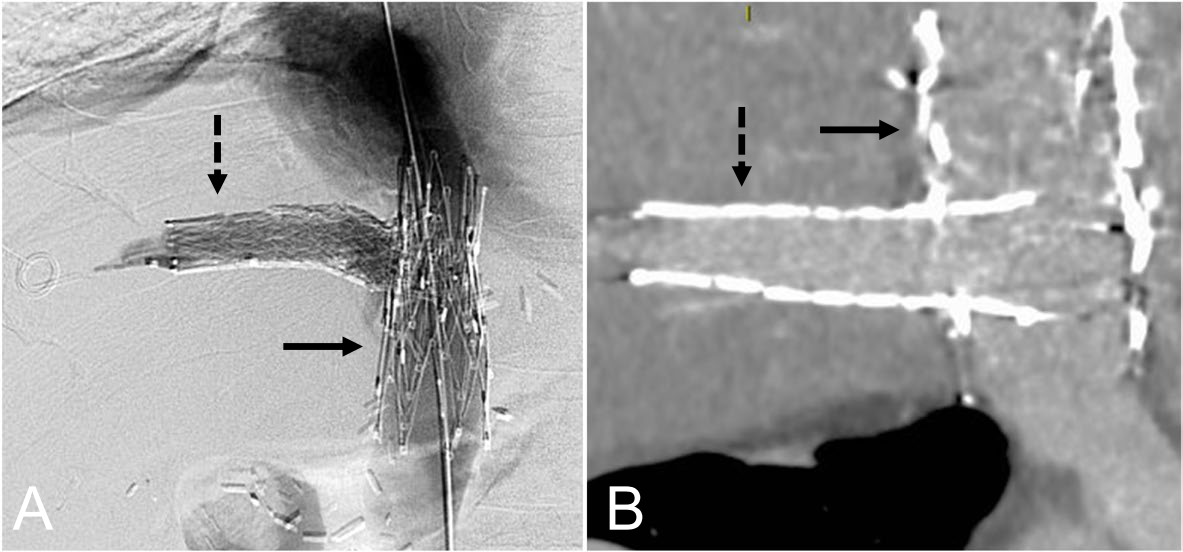
48-year-old male with hepatitis C cirrhosis, status post hepatic transplantation with a piggyback anastomosis presented with ascites, and underwent bifurcated hepatocaval stent reconstruction with two overlapping Z-stents in the IVC [solid arrow] and a Venovo stent in the right hepatic vein [dotted arrow] as seen on diagnostic venography (**A**) and contrast-enhanced CT (**B**)

This report describes the bifurcated hepatocaval T-stent reconstruction technique for seven OLT patients with symptomatic HVOO (patient demographics in Table 1). For most patients, two stents were placed while for two patients, three stents were placed (stent and procedure details in Table 2). One HV stent was replaced 21 days after the intervention. The approach involves sequential deployment of interlocking stents: an IVC stent is placed in the supra- and intrahepatic IVC across the level of the piggyback anastomosis [3]. The hepatic vein was then selected through the interstices of the IVC stent, and a second stent was placed through the IVC stent in a T configuration.

**Table 1:** Patient demographics

Subject Number	Age, Sex	Liver Disease Etiology	Liver Transplant Date, anastomosis type	Interval to intervention	Prior intervention	Indication for procedure	Diagnostic imaging
1	69, M	Alcoholic Cirrhosis and HCC	2004, piggyback	1 month	no	Ascites, LFTs	US w doppler
2	52, M	HCV and Alcoholic Cirrhosis	2006, piggyback	10 months	Angioplasty of IVC and HV	Ascites and LFTs	US w doppler
3	53, F	HCV	2009, piggyback	1.5 months	no	Abnormal LFTs	US w doppler
4	58, M	HCV and Alcoholic Cirrhosis	2008, piggyback	13 months	HV angioplasty	Ascites and LFTs	US w doppler
5	65, M	HCV and Alcoholic Cirrhosis	1994, piggyback	265 months	no	Ascites	CTAP w contrast
6	48, M	HCV	2007, piggyback	145 months	IVC angioplasty on 4 separate occasions	Ascites	US w doppler
7	64, F	Alcoholic Cirrhosis	2017, piggyback	25 months	no	Ascites	US w doppler

*HCV* Hepatitis C Virus, *HBV* Hepatitis B Virus, *HCC* Hepatocellular Carcinoma, *LFTs* Liver Function Tests

**Table 2:** Stent and Procedure Details

Subject Number	Sedation	Stenosis type: IVC, HV, or both?	Stent Details	Pre-procedural gradients	Post procedural gradient	Technical success, immediate complications
1	Moderate sedation	both	HV: 12 mm × 40 mm Wallstent IVC: 18 mm × 40 mm Wallstent	RA pressure: 4 mmHg Free HV: 25 mmHg Wedged HV: IVC: 17 mmHg	IVC-RA: 12 mmHg	Yes, no
2	Moderate sedation	both	HV: 12 mm × 40 mm Wallstent IVC: 14 mm × 40 mm Wallstent	Not recorded	Not recorded	Yes, no
3	Moderate sedation	Both	HV: Two 10 mm × 20 mm Wallstents IVC: 20 mm × 45 mm Wallstent	RA pressure: 5–6 mmHg Free HV: 12–13 mmHg Wedged HV: Infrahepatic IVC: 8–9 mmHg	Right hepatic vein: 14–15 mmHg; right atrium: 11–12 mmHg Intra-stent IVC: 10–12 mmHg; right atrium: 8–9 mmHg	Yes, no
4	Moderate sedation	Both	HV: 14 mm × 40 mm Nitinol Smart stent IVC: 22 mm × 45 mm Wallstent	RA pressure: 12 mmHg Free HV: 21 mmHg Wedged HV: IVC: 20 mmHg	RA pressure: 6 mmHg Free HV: 8 mmHg Wedged HV: IVC:	Yes, no
5	General anesthesia	IVC	HV: 14 × 40 mm Smart stent IVC: 25/30 mm × 120 mm Wallflex stent	Not recorded	RA: 10 mmHg RHV: 12 mmHg Infrarenal IVC: 12 mmHg, Lower IVC stent: 11–12 mmHg, Upper IVC stent 10–11 mmHg	Yes, no
6	Moderate sedation	Both	HV: 14 mm × 40 mm Venovo stent IVC: Two 25 mm × 50 mm Gianturco Z-stents	Not recorded	Not recorded	Yes, no
7	General anesthesia	Both	HV: 16 mm × 60 mm Venovo stent IVC: 25 mm × 50 mm Gianturco Z-stent	RA pressure: 2 mmHg Free HV: 10 mmHg Wedged HV: 14 mmHg IVC: 7 mmHg	RA pressure: 8 mmHg Free HV: 12 mmHg Wedged HV: not recorded Suprarenal IVC: 8 mmHg	Yes, no

The T configuration approach was technically successful in all seven patients with no periprocedural complications observed (Table 3). Technical success was defined as the successful venous reconstruction with bifurcated stents of the hepatocaval venous outflow obstruction with maintenance of venous patency. However, one HV stent migration occurred 21 days post-procedure, requiring retrieval and replacement. Clinical outcomes were encouraging, with five of seven patients (71%) achieving either complete resolution(*n* = 3) or significant improvement (*n* = 2) of symptoms at a mean follow up of 48 days. The remaining two patients did not achieve clinical success with both patients requiring angioplasty within two months of the intervention. One of these patients needed an additional IVC stent placement for in-stent stenosis. Continued lack of improvement prompted additional treatments with a long-term Denver shunt in one patient, and a transjugular intrahepatic portosystemic shunt (TIPS) in the other. Patient follow-up included imaging to assess patency and flow with Doppler Ultrasound (*n* = 5) or CT venography (*n* = 2). 100% of stents placed were patent at follow-up with a mean follow-up duration of 1,737 days and a range of 32 – 4,677 days. These results compare favorably with prior studies on isolated IVC stenting: Donaldson et al. [4] observed a 96% stent patency rate (113/118 stents) at last follow-up with symptom relief in 85% of patients. Ko et al. [5] found 91% of patients (20/22) needed no further interventions to restore patency at a mean follow up period of 42 weeks.

**Table 3:** Clinical Outcomes

Subject Number	Clinical success, when (days post procedure)	Postprocedural Complications	Repeat intervention	Stent patent at last f/u imaging (days), stent lack of migration (days)	Final Clinical Outcome
1	Yes, 22	No	no	56 (patient died), 51	Improved ascites control, pt died of complication of graft rejection
2	No: required long-term denver-shunt for ascites control, N/A	No	Angioplasty and additional IVC stent placed 2 months after procedure for stenosis in the IVC at the superior border of the first IVC stent. Denver shunt 2 months post procedure	4,677, 4,677	Dependent on long-term use of Denver shunt for ascites control. Alive
3	Yes, 91	Yes, stent migration 21 days post-intervention: one HV stent migrated into the IVC	No	3,798, 3436	Ascites and lower extremity edema resolved, alive
4	No: required TIPS for ascites control, N/A	No	Re-angioplasty of HV at 1 and 12 months post procedure. TIPS performed 15 months post procedure	2,598, 638	Required TIPS for resolution of symptoms, alive
5	Yes, 44	No	no	874, 43	Ascites and lower extremity edema resolved, alive
6	Yes, 42	No	no	126, 126	Ascites and lower extremity edema resolved, alive
7	Yes, 39	No	no	32, 71	Decrease in ascites, alive

*TIPS* Transjugular Intrahepatic Portosystemic Shunt

The bifurcated T-stent configuration offers several theoretical advantages over conventional approaches. First, by preserving at least one patent HV through intentional stent interposition, it prevents the “jailing” of hepatic outflow that can occur with IVC stent placement alone. Second, the interlocking design provides mutual stent stabilization, reducing risk of stent migration, which is a serious complication that may require surgical intervention [1]. One patient did experience a stent migration event despite the T-stent configuration, but it was likely due to the type of stent used and the position within the HV. A rigid, braided design from Wallstent was used while the authors have found that a non-braided, self-expanding stent such as Venovo or SMART has produced better results. Precise stent placement, stent size and stent properties all play crucial roles in maintaining stability and integrity of the T-stent configuration.

The T-stent configuration has been formally described for use in the post liver transplant setting [2]. While current literature has focused on the use of a singular stent, the bifurcated technique is a technically complex procedure and requires the precise deployment of multiple stents. Other techniques to reduce IVC stent migration have been described in the literature including oversizing stents and using a percutaneous T-fastener insertion to anchor stents in palliation of malignant IVC stenosis complicated with repeat stent migration [6, 7]. In the case of the T-fastener, a percutaneous, gastrostomy-type T-fastener was placed transhepatically under fluoroscopic guidance into the IVC lumen through interstices of the IVC stent to anchor the stent in place for palliation of malignant IVC stenosis complicated by repeat stent migration [7]. The potential for future studies is significant; rigorous research is needed to allow for the refinement of patient selection criteria, and to evaluate T-stenting against traditional stenting methods to determine the most effective treatment modality for various patient populations. Further investigation into optimal stent designs, materials, and deployment techniques can reduce procedural complications and improve patient outcomes. Finally, understanding how T-stenting can impact subsequent procedures such as re-transplantation or imaging studies is crucial to long-term and comprehensive patient care.

In summary, the T-stent technique provides a valuable alternative for managing symptomatic HVOO in OLT recipients. Its high technical success rate, durable patency, and theoretical advantages over conventional stenting merit further investigation through larger, prospective studies. Future research should focus on optimizing protocols while directly comparing outcomes with existing stenting approaches.

## Data Availability

All data generated or analysed during this study are included in this published article and its supplementary information files.

## Abbreviations

OLT: Orthotopic liver transplant
HVOO: Hepatic venous outflow obstruction
IVC: Inferior vena cava
HV: Hepatic vein
